# The Extended Linguistic Hellwig’s Methods Based on Oriented Fuzzy Numbers and Their Application to the Evaluation of Negotiation Offers

**DOI:** 10.3390/e24111617

**Published:** 2022-11-06

**Authors:** Ewa Roszkowska, Tomasz Wachowicz, Marzena Filipowicz-Chomko, Anna Łyczkowska-Hanćkowiak

**Affiliations:** 1 Faculty of Computer Science, Bialystok University of Technology, Wiejska 45A, 15-351 Bialystok, Poland; 2 Department of Operations Research, University of Economics in Katowice, 1 Maja 50, 40-287 Katowice, Poland; 3 Institute of Economics and Finance, WSB University of Poznan, ul. Powstańców Wielkopolskich 5, 61-895 Poznan, Poland

**Keywords:** fuzzy multiple criteria decision making, fuzzy preference information, linguistic evaluation of negotiation offers, oriented fuzzy number, negotiation scoring system, preference analysis

## Abstract

This study proposes a novel fuzzy framework for eliciting and organizing the preference information of the negotiator to allow for the evaluation of negotiation offers. The approach is based on verbal evaluation of negotiation options that operates with linguistic variables to handle vague preferences and operationalizes them through oriented trapezoidal fuzzy numbers. Two variants of the linguistic method based on Hellwig’s approach and oriented fuzzy numbers are proposed, which can be applied to building a scoring system for the negotiation template. Then, an example of determining such a scoring system and using it to evaluate the negotiation offers in typical multi-issue negotiation is shown. The results are discussed and compared with other methods known from the literature, in which the preference information is organized similarly but processed differently. The comparison shows that the presented methods can be an alternative to Simple Additive Weighting or TOPSIS methods that may also operate with oriented fuzzy numbers, but some of their characteristics may be problematic from the viewpoint of data interpretation. The former requires defuzzification of the global scores determined, while the latter requires the compulsory use of two reference points derived mechanically out of the negotiation space. By applying modified Hellwig’s approaches, the former and the latter may be easily avoided.

## 1. Introduction

Negotiation is the process of exchanging offers, concessions, and argumentation, where conflicting issues need to be evaluated [1]. In the pre-negotiation phase, negotiators must structure the negotiation problem, build the negotiation template, elicit their preferences and build negotiation scoring systems. Such scoring systems help in the further negotiation process in evaluating offers, measuring the scale of concessions, and estimating negotiation progress. Finally, in the post-negotiation phase, they can be used to search for fair and satisfying final solutions or improvements to the negotiated agreement [2].

The evaluation of negotiation offers is possible if each party thoroughly analyzes their preferences during the pre-negotiation phase. Such an analysis requires mapping the preference information expressed by the decision maker, usually verbally, in the direct discussion with an analyst or facilitator, into the system of corresponding quantitative meanings. Then, this quantitative preference information needs to be organized and processed according to the previously recognized DM’s individual preference model. The latter should be done particularly diligently to ensure that the scoring system and scoring formulas reliably copy the DM’s intrinsic behavior in using their preferences for evaluating the negotiation offers. Therefore, such a preference analysis is usually conducted with the support of various multiple criteria decision-making (MCDM) or fuzzy multiple criteria decision-making (FMCDM) methods, as from the viewpoint of individual negotiator analyzing preferences for multi-issue negotiation resembles analyzing the preferences in any single-DM MCDM problem [2,3,4]. The choice between multiple criteria techniques depends on the negotiation problem, types of issues, available information, and properties of the multiple criteria technique, among others. MCDM methods are particularly useful where the negotiation problem is well structured, i.e., the issues and options can be precisely specified while defining the negotiation problems, and their evaluations (e.g., weights) can be measured using crisp numbers. The MCDM methods used to determine the scoring systems in such situations are DR (Direct Rating) [5], AHP (Analytic Hierarchy Process) [6], TOPSIS (Technique for Order Preferences by Similarity to Ideal Solution) [7], MARS (Measuring Attractiveness near Reference Situations) [8,9], UTA (UTilités Additives) [10], ELECTRE (ÉLimination Et Choix Traduisant la Realité) [11], among others. Within this group of MCDM methods, especially vital are those based on a reference point (ideal solution) or two reference points (ideal and anti-ideal solutions) such as TOPSIS, VIKOR (Serb. Vlse Kriterijumska Optimizacija i Kompromisno Resenje) [12], and BIPOLAR [13]. The ideal solution can correspond with the aspiration level defined in pre-negotiations, while the anti-ideal solution does with the reservation level [14]. On the other hand, FMCDM can be applied in the ill-structured negotiation problem where the negotiators express ratings and criteria weights imprecisely, subjectively, or vaguely. Such imprecise evaluations can result from the lack of information, measurement error, cognitive limitations, or subjective evaluation of the options, which are often observed in real-life negotiation [15]. Applications of fuzzy multiple criteria decision-making methods to determine the scoring systems can be found in many papers, e.g., [7,15,16,17].

In some situations, it may be helpful and more flexible to operate with the linguistic evaluation of options and offers and describe preferences naturally and intuitively, e.g., when qualitative negotiation issues need to be considered. An example of such a variable in typical business negotiations may be the returns policy, terms of warranty, or quality. The numerical values may also be evaluated through linguistic variables if the granularity of such an evaluation is sufficient for the supported negotiator. This granularity, i.e., the cardinality of the linguistic term set used in preference declarations, should be small enough not to provide the negotiator with too many evaluation options to declare (a useless precision). On the other hand, it should be rich enough to allow the discrimination of the assessments in a limited number of degrees.

The linguistic values can be represented in various ways, e.g., through fuzzy sets [18,19], intuitionistic fuzzy sets [20], or ordered fuzzy sets [17]. The linguistic approach in negotiation preference elicitation and support has been considered so far in a few papers (see, e.g., [17,21]). In paper [17], the scale of values used in evaluating the negotiation options included the following expressions: very bad, bad, average, good, and very good together with intermediate values such as “at least good” or “at most good” and was represented by oriented fuzzy numbers (OFNs). This scale was used to verify the applicability of the Oriented Fuzzy SAW (OF-SAW) and Oriented Fuzzy TOPSIS (OF-TOPSIS) methods based on oriented fuzzy numbers in scoring negotiation offers.

The motivation for this study is the following. We want to take advantage of oriented fuzzy numbers represented in linguistic terms for dealing with unprecise information in evaluating negotiation offers presented in [17]. The second motivation is applying Hellwig’s framework, a progenitor to TOPSIS and VIKOR, for building a negotiation scoring system. Two variants of the extended linguistic Hellwig’s method, i.e., Oriented Fuzzy Hellwig’s methods (OF-Hellwig’s), are presented and compared. The first one, named OF-H1, uses one reference point, and the other, OF-H2, operates with two reference points. Although various researchers have earlier proposed many modifications of both variants of Hellwig’s method (see Tables 2 and 3), this paper’s novelty is the application of oriented fuzzy numbers in the modified Hellwig’s measure and their use to rank-ordering negotiation offers. We also compare these methods with OF-TOPSIS and OF-SAW in an illustrative example. The TOPSIS method is based on the concept that the chosen alternative should be the closest to the positive ideal solution and the farthest from the negative ideal solution [22]. On the contrary, the first variant of classical Hellwig’s method takes into account the distance to the positive ideal solution only [23], while in the second variant, the distances between the best (positive ideal) and the worst (negative ideal solution) solutions are used in the normalizing measure.

The advantages of this new approach are the following:

- It allows for linguistic evaluation negotiation offers;
- The scoring procedure implemented does not need the normalization procedure for options;
- It allows using either one or two reference points;
- In OF-H1, OF-H2 procedure rank reversal can be avoided when new offers are added to the evaluation process; additionally, OF-H2  also avoids changing scores points;
- Both Hellwig’s methods based on oriented fuzzy numbers are intuitive and easy tools for rank ordering negotiation offers and can be alternatives to the methods presented in [17], i.e., OF-TOPSIS and OF-SAW.

The remainder of this paper is structured as follows. In Section 2, reviews of oriented fuzzy sets and linguistic approach are briefly outlined. The classical Hellwig’s methods (H1 and H2) and their extended variants based on a linguistic approach represented by oriented fuzzy numbers (OF-H1 and OF-H2) are presented in Section 3. The case study and implementation of the proposed method for evaluating negotiation offers are then presented in Section 4. It additionally provides a discussion and comparative analyses of the proposed method to the current approaches showing the advantages and disadvantages of the former in Section 5. In Section 6, concluding remarks and future research directions are presented.

## 2. Introducing the Oriented Fuzzy Numbers (OFN)

### 2.1. Basic Notions

Fuzzy numbers are an approximation of imprecise numbers. They were introduced in 1978 by Dubois and Prade [24] as a subset of the fuzzy real line. The ordered fuzzy numbers, introduced by Kosiński [25], are an extension of fuzzy numbers that cope with the problems of increasing imprecision when simple arithmetic calculations are performed. A formal description of the Ordered Fuzzy Number model, motivation for building it, and some applications can be found in [25,26,27,28]. The Ordered Fuzzy Number model was defined as an ordered pair of continuous functions $\left(f_{A},\;g_{A}\right)$ with the orientation which provides additional information about a fuzzy number. To honor the contribution of Witold Kosiński in the development of the considered model, the numbers are also named Kosiński’s Fuzzy Numbers [27].

**Definition** **1** **[26].** *The ordered fuzzy number $A=\left(f_{A},\;g_{A}\right)$ is an ordered pair of continuous functions $f_{A},\;g_{A}\;:\left[0,\;1\right]\;\to \;R$, called the up part, and the down part of A, respectively.*

Distinction between pairs $\left(f_{A},\;g_{A}\right)$ and $\left(g_{A},\;f_{A}\right)$ introduces additional information (orientation), which allows to analyze whether a given observed imprecise value is generally likely to increase or decrease. One possibility is interpreting orientation as a trend of fuzzy observation or measurement [25,27,29].

In recent years, ordered fuzzy numbers (Kosiński’s numbers) have been more and more often used to describe and analyze various decision-making problems [27]. In particular, Roszkowska and Kacprzak proposed the fuzzy SAW and fuzzy TOPSIS procedures based on ordered fuzzy numbers [30]. These methods were extended by Kacprzak to the case of group decision-making in [31,32].

The important drawback of Kosiński’s theory is that the space of ordered fuzzy numbers is not closed under Kosiński’s addition; thus, there exist such ordered fuzzy numbers which are not fuzzy numbers (named as improper ordered fuzzy numbers). In 2018, Piasecki revised Kosiński’s theory so that the space of ordered fuzzy numbers is closed under proposed arithmetic operations [33]. To distinguish between ordered fuzzy numbers and fuzzy numbers with modified operations, they were named Oriented Fuzzy Numbers (OFNs). Importantly, results obtained using revised arithmetic operations are the best approximation of results obtained using Kosiński arithmetic operations. Thus, in a situation with proper ordered fuzzy numbers, those operations provide this same result. The differences between fuzzy numbers, Kosiński numbers, and oriented fuzzy numbers have been discussed in detail by Piasecki and Łyczkowska-Hanćkowiak [33,34]. The OFNs were used in economics and finance for evaluation of the process of the assessment of the credit standing of the potential borrower [35,36], for modeling Japanese candles [37], for present value evaluation under the impact of behavioral factors [38], in portfolio analysis [39] or imprecise investment recommendations [40].

The applications of the TOPSIS technique based on oriented fuzzy numbers (OF-TOPSIS) to support the evaluation of negotiation offers have been studied by Piasecki and Roszkowska [17]. In other papers, the problems of the SAW technique based on oriented fuzzy numbers (OF-SAW) were discussed, especially the fuzzy ranking of evaluated alternatives [21], the impact of the orientation of the ordered fuzzy assessment on the OF-SAW method, application of the OF-SAW method in credit risk assessment [35,36].

### 2.2. Trapezoidal Oriented Fuzzy Numbers (TrOFNs)

A special case of an oriented fuzzy number (which we will use in this paper) is a trapezoidal oriented fuzzy number (TrOFN) defined in the following way.

**Definition** **2** **[33].** *For any monotonic sequence $\;\left\{a,b,c,d\right\}\subset \mathbb{R}$, the trapezoidal oriented fuzzy number (TrOFN) $\overset{↔}{Tr}\left(a,b,c,d\right)$ is determined explicitly by its membership functions $\mu_{Tr}\left(\cdot |a,b,c,d\right)\in {\left[0,1\right]}^{\mathbb{R}}$as follows:*

**Figure 1:**
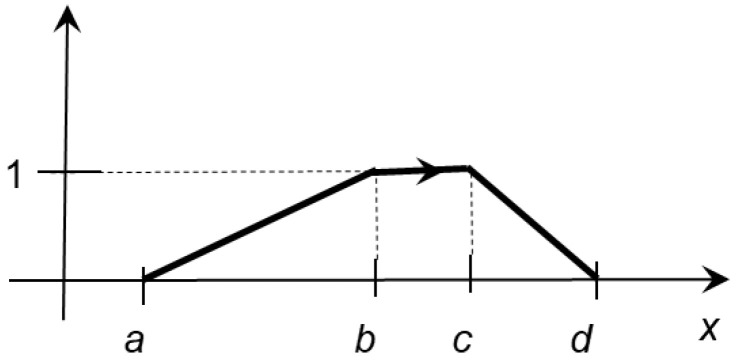
A positively oriented TrOFN $\overset{↔}{Tr}\left(a,b,c,d\right)$.

**Figure 2:**
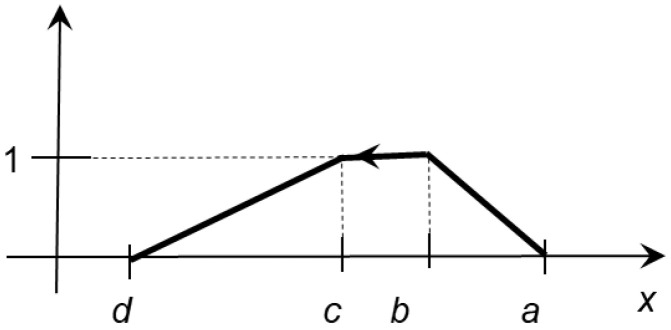
A negatively oriented TrOFN $\overset{↔}{Tr}\left(a,b,c,d\right)$.

*(a)* 
(1) $$\mu_{Tr}\left(x|a,b,c,d\right)=\left\{\begin{array}{c} \;0,\;\;\;\;\;\;\;\;\;\;\;\;\;\;\;\;\;\;\;\;\;\;\;\;\;\;\;\;\;\;\;x\notin \left[a,d\right],\\ \frac{x-a}{b-a},\;\;\;\;\;\;\;\;\;\;\;\;\;\;\;\;\;\;\;\;\;\;\;x\in \left[a,b\right],\;\\ 1,\;\;\;\;\;\;\;\;\;\;\;\;\;\;\;\;\;\;\;\;\;\;\;\;\;\;\;\;\;\;\;x\in \left[b,c\right],\;\\ \frac{x-d}{c-d},\;\;\;\;\;\;\;\;\;\;\;\;\;\;\;\;\;\;\;\;\;\;\;x\in \left[c,d\right], \end{array}\right.\;\;\;\mathrm{if}\;a<d\;,$$
  *and such a trapezoidal oriented fuzzy number* $\overset{↔}{Tr}\left(a,b,c,d\right)$ *is positively oriented. The additional information is marked graphically with arrows (see* Figure 1 *).*
*(b)* 
(2) $$\mu_{Tr}\left(x|a,b,c,d\right)=\left\{\begin{array}{c} \;0,\;\;\;\;\;\;\;\;\;\;\;\;\;\;\;\;\;\;\;\;\;\;\;\;\;\;\;\;\;\;\;x\notin \;\left[d,a\right],\\ \frac{x-d}{c-d},\;\;\;\;\;\;\;\;\;\;\;\;\;\;\;\;\;\;\;\;\;\;\;x\in \left[d,c\right],\\ 1,\;\;\;\;\;\;\;\;\;\;\;\;\;\;\;\;\;\;\;\;\;\;\;\;\;\;\;\;\;\;\;x\in \left[c,b\right],\\ \frac{x-a}{b-a},\;\;\;\;\;\;\;\;\;\;\;\;\;\;\;\;\;\;\;\;\;\;\;\;x\in \left[b,a\right]. \end{array}\right.\;\;\mathrm{if}\;d<a,\;$$
  *and such trapezoidal oriented fuzzy number* $\overset{↔}{Tr}\left(a,b,c,d\right)$ *is negatively oriented* *(see* Figure 2 *).*
*(c)*  *If* a=d *, then TrOFN* $\overset{↔}{Tr}\left(a,a,a,a\right)$ *represents a crisp number* a∈ℝ *, which is not oriented.*

The space of all trapezoidal OFNs is denoted by $K_{Tr}$. In the set of all trapezoidal oriented fuzzy numbers $K_{Tr}$, the operations of generalized addition ⊞ and multiplication by the real number ⊡ are defined below.

**Definition** **3** **[33].** *For any pair $\left(\overset{↔}{Tr}\left(a,b,c,d\right),\overset{↔}{Tr}\left(p-a,q-b,r-c,s-d\right)\right)\in K_{Tr}^{2}$ and β∈ℝ:*

(3) $$\overset{↔}{Tr}\left(a,b,\;c,d\right)⊞\overset{↔}{Tr}\left(p-a,q-b,\;r-c,s-d\right)=\;=\left\{\begin{array}{c} \overset{↔}{Tr}\left(\min\left\{p,q\right\},q,\;r,\max\left\{r,s\right\}\right),\;\;\;\;\;\;\;\;\;\left(q<r\right)\vee \left(q=r\wedge p\leq s\right),\;\;\\ \;\overset{↔}{Tr}\left(\max\left\{p,q\right\},q,\;r,\min\left\{r,s\right\}\right),\;\;\;\;\;\;\;\;\left(\;q>r\right)\vee \left(q=r\wedge p>s\right). \end{array}\right.\;$$

(4) $$\beta ⊡\overset{↔}{Tr}\left(a,b,c,d\right)=\overset{↔}{Tr}\left(\beta \cdot a,\beta \cdot b,\beta \cdot c,\beta \cdot d\right).$$

It is worth noting here that for any pair $\left(\overset{↔}{Tr}\left(a,b,c,d\right),\overset{↔}{Tr}\left(e,f,g,h\right)\right)\in K_{Tr}^{+}\times K_{Tr}^{+}\cup K_{Tr}^{-}\times K_{Tr}^{-}$ the calculations are much simpler, i.e.,

(5) $$\overset{↔}{Tr}\left(a,b,\;c,d\right)⊞\overset{↔}{Tr}\left(e,f,\;g,h\right)=\overset{↔}{Tr}\left(a+e,b+f,\;c+g,d+h\right),$$

where $K_{Tr}^{+}$ ($K_{Tr}^{-})$ denotes a set of positively (negatively) oriented trapezoidal fuzzy numbers.

The formal differences between fuzzy numbers and oriented fuzzy numbers, especially the operations of addition and multiplication by the real numbers, were investigated in [34].

**Definition** **4** **[17].** *The distance between any two trapezoidal numbers $\overset{↔}{Tr}\left(a,b,c,d\right)$ and $\overset{↔}{Tr}\left(e,f,g,h\right)$ is determined using the formula*

(6) $$d\left(\overset{↔}{Tr}\left(a,b,\;c,d\right),\;\overset{↔}{Tr}\left(e,f,g,h\right)\right)=\sqrt{{\left(a-e\right)}^{2}+{\left(b-f\right)}^{2}+{\left(c-g\right)}^{2}+{\left(d-h\right)}^{2}}.$$

When fuzzy numbers are used in decision support, defuzzifying them to conduct some specific analysis is often required. In our paper, we use the notion of a defuzzification technique based on TrOFN.

**Definition** **5** **[17].** *Defuzzification functional is the map $\phi :K_{Tr}\cdot \mathbb{R}$ that for any monotonic sequence $\;\left\{a,b,c,d\right\}\subset \mathbb{R}$ satisfies the following conditions:*

(7) $$\min\left\{a,b,c,d\right\}\leq \phi \left(\overset{↔}{Tr}\left(a,b,c,d\right)\right)\leq \max\left\{a,b,c,d\right\},$$

(8) $$\forall_{r\in \mathbb{R}}:\;\;\;\;\;\;\phi \left(\overset{↔}{Tr}\left(a,b,c,d\right)⊞\overset{↔}{Tr}\left(r,r,r,r\right)\right)=\phi \left(\overset{↔}{Tr}\left(a,b,c,d\right)\right)+r,$$

(9) $$\forall_{r\in \mathbb{R}}:\;\;\;\;\;\;\phi \left(r⊙\overset{↔}{Tr}\left(a,b,c,d\right)\right)=r\cdot \phi \left(\overset{↔}{Tr}\left(a,b,c,d\right)\right).$$

Various defuzzification methods are known and used in the theory and practice of fuzzy numbers [17,29,30,41]. Some examples of them (used later in the empirical part of this paper) are presented in Table 1.

### 2.3. Linguistic Variables

The linguistic variable is a variable whose values are not numbers but words or sentences expressed in a natural or artificial language [42,43,44]. The linguistic analysis, variable transformation methodologies, and applications of the linguistic approach in decision-making are summarized in [18,19,45,46,47]. According to Herrera and Herrera-Viedma [19], the linguistic value is characterized by a label with a semantic value which is an expression belonging to a given linguistic term set and mechanism for generating the linguistic descriptors. When the meaning of a semantic value is imprecise, then the labels from the applied linguistic term set can be represented by fuzzy numbers.

The scale of fuzzy numbers should be adopted considering a particular situational context of the decision-making problem and its subjective interpretation by the decision-maker (DM), so different numerical scales of linguistic terms for different DMs can be derived [19,48,49]. Here, we recall the linguistic scale based on OFNs presented in earlier studies [17,30] and show a possibility of extending this scale.

First, the Tentative Order Scale (TOS) with five levels is defined as follows: $TOS=\left\{V_{1};\;V_{2};\ldots ;V_{5}\right\},\;$where $V_{1}=\mathrm{Very}\;\mathrm{Bad}\;\left(VB\right),\;V_{2}=\mathrm{Bad}\;\left(B\right),\;V_{3}=\mathrm{Average}\;\left(A\right),\;V_{4}=\mathrm{Good}\;\left(G\right)$, $V_{5}=\mathrm{Very}\;\mathrm{Good}\;\left(VG\right)$. Each reference point $V_{j}$ is equivalent to the numerical diagnosis  j∈ℕ.

Next, TOS may be extended by introducing intermediate values “at least”, described by the symbol L, and “at most”, described by the symbol M. The linguistic term $L.V_{j}$ means “no worse than $V_{j}$ and worse that $\;V_{j+1}$” and $M.V_{j}$ means “no better than $V_{j}$ and better that$\;V_{j-1}$”. Moreover, it was assumed that the $L.V_{j}$ “is better than” $M.V_{j}$. The numerical representation of the phrase “at least” is denoted by the GE and expression $L.V_{j}$ is equivalent to numerical diagnosis GE.j. Consequently, the numerical representation of the phrase “at most” is denoted by the LE and expression $M.V_{j}$ is equivalent to numerical diagnosis  LE.j. The extended Order Scale has the following form:

(10) $$OS=\left\{VB;\;L.VB;M.B;\;B;L.B;M.A;\;A;\;L.A;\;M.G;G;L.G;\;M.VG;\;VG\right\}.$$

The Numerical Diagnosis Set corresponding with OS has the following form:

(11) $$ND=\left\{1;\;GE.1;LE.2;\;2;GE.2;LE.3;\;3;\;GE.3;\;LE.4;4;GE.4;\;LE.5;\;5\right\}.$$

The numerical diagnosis  j∈ℕ is represented by TrOFN in the following way:

(12) $$\mathrm{for}\;j=1,2,\ldots ,5\;j\cdot {\overset{↔}{X}}_{j}=\overset{↔}{Tr}\left(j,j,j,j\right).$$

All numerical diagnoses GE.j and LE.j are imprecise. Therefore, we determine the converting system, which transforms numerical diagnoses into performance ratings described by TrOFN in the following way:

(13) $$\mathrm{for}\;j=2,3,4,5\;\;\;\;\;\;\;LE.j\cdot {\overset{↔}{X}}_{Lj}=\overset{↔}{Tr}\left(j,j,j-\frac{1}{2},j-1\right);$$

(14) $$\mathrm{for}\;j=1,2,3,4\;\;\;\;\;\;\;GE.j\cdot {\overset{↔}{X}}_{Gj}=\overset{↔}{Tr}\left(j,j,j+\frac{1}{2},j+1\right).$$

In this way, we obtain the following Numerical Order Scale:

(15) $$NOS=\left\{{\overset{↔}{X}}_{j}:j=1,2,\ldots ,5\right\}\cup \left\{{\overset{↔}{X}}_{Lj}:j=2,3,4,5\right\}\cup \left\{{\overset{↔}{X}}_{Gj}:j=1,2,3,\;4\right\},$$

determined by trapezoidal OFN.

Let us observe that the orientation of OFN represents the relations *LE* (negative orientation) and *GE* (positive orientation). The consolidated mechanism of transformation of considered linguistic values into performance ratings expressed on *NOS* is summarized in Table 2.

**Figure 3:**
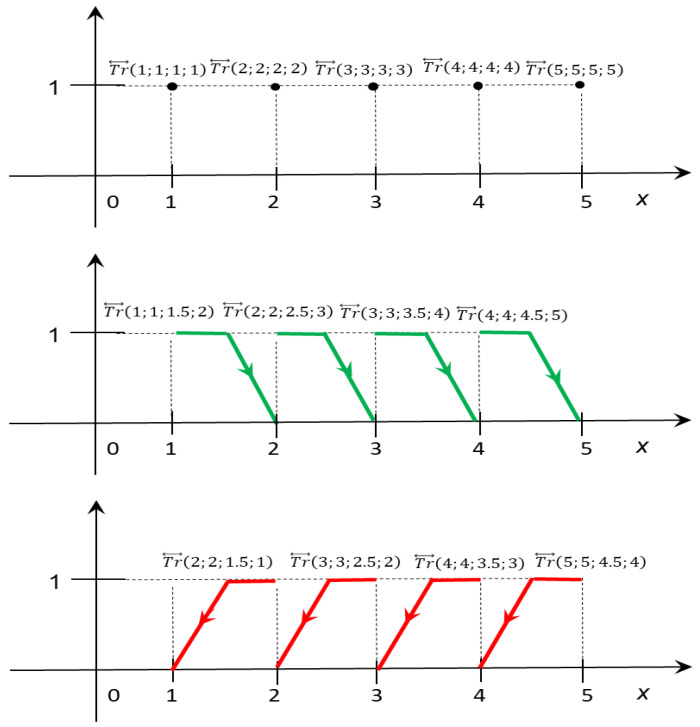
Graphical representation of linguistic scale. Different types of linquistic values are marked by colour.

The graphical representation of the linguistic scale is presented in Figure 3.

Let us note that the scale can be extended in different ways. For instance, we can introduce an additional level of values meaning “above”, and described by the symbol A. The linguistic term $A.V_{j}$ means “better than $V_{j}$ and worse that$\;V_{j+1}$”. The numerical representation of the phrase “above” is denoted by the ANE. The expression $A.V_{j}$ is equivalent to numerical diagnosis ANE.j. We can transform numerical diagnoses into performance ratings described by TrOFN in the following way:

(16) $$\mathrm{for}\;j=1,2,3,4\;\;\;ANE.j.⟶{\overset{↔}{X}}_{Lj}=\overset{↔}{Tr}\left(j,j,j+\frac{1}{2},j+\frac{3}{4}\right).$$

## 3. The Fuzzy Linguistic Hellwig’s Methods Based on Fuzzy Oriented Numbers

### 3.1. The Classical Hellwig’s Procedure and Its Modifications

The classical Hellwig’s procedure was introduced in 1968 as a taxonomic method that allowed comparisons of the economic development of countries [23]. In 1972, it gained popularity in the international literature through the realization of the UNESCO research project on the human resources indicators for less developed countries [50]. The construction of synthetic measure in the original Hellwig’s procedure is based on the distances of objects from the abstract pattern of economic development (positive ideal solution). However, there is a second and less-known variant of Hellwig’s method, which in the scores aggregation procedure takes into account not only the ideal pattern but also the anti-pattern of development (negative ideal solution) [51]. This technique is close to the TOPSIS procedure [22], which is also based on two reference points. The TOPSIS basic concept is that the chosen alternative should have the shortest distance from the positive ideal solution (*PIS*) and the longest distance from the negative ideal solution (*NIS*). In contrast, in the second variant of Hellwig’s method, the distance between the positive and negative solutions is used to normalize the synthetic performance measure.

Over the years, the classical Hellwig’s procedure [23] was applied in many areas and modified for real data [52], fuzzy sets [53], intuitionistic fuzzy [54,55], and interval-valued fuzzy sets [56]. These applications and modifications are presented in Table 3.

The applications of the second variant of the Hellwig’s method [51] with two reference points and its modifications are presented in Table 4.

### 3.2. The Classical Variants of Hellwig’s Method

Let $C=\{C_{1},\ldots ,\;C_{n}\}$ be the set of criteria and $A=\{A_{1},\ldots ,\;A_{m}\}$ the set of alternatives. Let us assume that P and N are the sets of benefit and cost criteria, respectively $\left(C=P\cup N\right)$. Hellwig’s method consists of the following steps [23,51]:

**Step 1.** Define the data matrix:

(17) $$D=\left[\begin{array}{cccc} x_{11} & x_{12} & \cdots & x_{1n}\\ x_{21} & x_{22} & \cdots & x_{2n}\\ \vdots & \vdots & \ddots & \vdots \\ x_{m1} & x_{m2} & \cdots & x_{mn} \end{array}\right],$$

where $x_{ij}$ is the assessment of ith alternative with respect to the jth criterion $\left(i=1,2,\ldots ,m;\;j=1,2,\ldots ,n\right).$

**Step 2.** Determine the normalized data matrix:

(18) $$Z=\left[\begin{array}{cccc} z_{11} & z_{12} & \cdots & z_{1n}\\ z_{21} & z_{22} & \cdots & z_{2n}\\ \vdots & \vdots & \ddots & \vdots \\ z_{m1} & x_{m2} & \cdots & z_{mn} \end{array}\right]$$

using the standardization formula

(19) $$z_{ij}=\frac{x_{ij}-{\bar{x}}_{j}}{S_{j}},$$

where ${\overset{\cdot }{x}}_{j}=\frac{1}{m}\sum_{i=1}^{m}x_{ij}$, $S_{j}=\sqrt{\frac{1}{m}\sum_{i=1}^{m}{\left(x_{ij}-{\overset{\cdot }{x}}_{j}\right)}^{2}}$.

**Step 3.** Define the ideal solution (pattern of development) $O^{+}=\left[z_{1}^{+},z_{2}^{+},\ldots ,z_{n}^{+}\right]$ following the principle

(20) $$z_{j}^{+}=\left\{\begin{array}{ccc} \underset{i}{\max}z_{ij} & \mathrm{if} & z_{ij}\in P,\\ \underset{i}{\min}z_{ij} & \mathrm{if} & z_{ij}\in N. \end{array}\right.$$

**Step 4.** Calculate the distance of the ith alternative from the ideal solution using the Euclidean distance:

(21) $$d_{i}^{+}=\sqrt{\sum_{j=1}^{n}{\left(z_{ij}-z_{j}^{+}\right)}^{2}}.$$

**Step 5.** Calculate the synthetic measure for the ith alternative using one of the following formulas:

(22) $$H1\left(A_{i}\right)=1-\frac{d_{i}^{+}}{d_{0}},$$

where: $d_{0}=\overset{-}{d}+2S$, $\overset{-}{d}=\frac{1}{m}\sum_{i=1}^{m}d_{i}^{+}$, $S=\sqrt{\frac{1}{m}\sum_{i=1}^{m}{\left(d_{i}^{+}-\overset{-}{d}\right)}^{2}}$

Or

(23) $$H2\left(A_{i}\right)=1-\frac{d_{i}^{+}}{d^{+-}},$$

where $d^{+-}=\sqrt{\sum_{j=1}^{n}{\left(z_{j}^{+}-z_{j}^{-}\right)}^{2}}$, and $z_{j}^{-}$ is the performance of the anti-pattern determined form the following formula:

(24) $$z_{j}^{-}=\left\{\begin{array}{ccc} \underset{i}{\max}z_{ij} & \mathrm{if} & z_{ij}\in N,\\ \underset{i}{\min}z_{ij} & \mathrm{if} & z_{ij}\in P. \end{array}\right.$$

**Step 6.** Ranking the alternatives according to the decreasing values of $\;H1\left(A_{i}\right)$ or $H2\left(A_{i}\right).$

The synthetic measures H1 and H2  usually take the values from the interval [0, 1]. The higher the values of the measures, the closer the object is to the ideal solution.

**Remark.**  *Let us notice that the classical Hellwig’s approach does not consider the weights of the criteria.*

### 3.3. The Extended Variants of Hellwig’s Method Based on Oriented Fuzzy Numbers

Let $C=\{C_{1},\ldots ,\;C_{n}\}$ be the set of criteria and $A=\{A_{1},\ldots ,\;A_{m}\}$ the set of alternatives. The OF-Hellwig’s method can be described in the following steps:

**Step 1.** Define the Order Scale OS with r  levels and the Numerical Order Scale NOS with performance ratings represented by TrOFNs, i.e., $OS=\{{\overset{↔}{X}}_{1},\ldots ,{\overset{↔}{X}}_{r}\}$, where ${\overset{↔}{X}}_{k}=\overset{↔}{Tr}\left(k,\;k,\;k,\;k\right)$ for k=1,2,…r.

**Step 2.** Define the criteria weights $w_{j}$ which describe the importance of each criterion $C_{j}\;\left(j=1,\ldots ,n\right)$, in the evaluation of the alternatives, where

(25) $$w_{1}+w_{2}+\ldots +w_{n}=1.$$

Taking into account the information about criteria weights, we can distinguish situations where weights are completely known, completely unknown, and partially known. In MCDM, several approaches are proposed for determining the weights of criteria, which can generally produce two types of weights: “subjective” and “objective” ones [69,70]. The subjective weights are determined from the preference information obtained from DM while the latter are derived from the decision-making matrix and calculated by solving some predefined mathematical models. The most known subjective criteria weights are rank ordering methods [71], the trade-off method [72], DR (Direct Rating) [73,74], and AHP (Analytic Hierarchy Process) [75], among others.

In the case where DM may not be well oriented with all the aspects of a problem and has limited expertise, the use of objective criteria weights becomes helpful. The most popular objective method is the Shannon entropy method, which expresses the relative intensities of criteria important to signify the average intrinsic information transmitted to the decision maker [22]. The entropy-based weights of criteria evaluate value by measuring the degree of differentiation. The higher the degree of dispersion of the measured value, the higher the degree of differentiation of the criterion, and more information can be derived. Therefore, a higher weight should be given to the criterion. Otherwise, such a criterion will be judged unimportant and a low weight should be assigned to the criterion. Several modifications of entropy-based methods in the fuzzy environment can be found in the literature [76,77,78].

**Step 3.** Evaluate each alternative $A_{i}\in A$ by the vector $\left({\overset{↔}{X}}_{i,1},{\overset{↔}{X}}_{i,2},\ldots ,{\overset{↔}{X}}_{i,n}\right)$, where ${\overset{↔}{X}}_{i,j}\;\in NOS$ is the performance (rating) of ith alternative with respect to jth criterion.

**Step 4**. Identify the *PIS* and the *NIS*, which are the following:

(26) $$PIS=({\overset{↔}{X}}_{r},\;{\overset{↔}{X}}_{r},\ldots ,{\overset{↔}{X}}_{r}\;),$$

(27) $$NIS=({\overset{↔}{X}}_{1},\;{\overset{↔}{X}}_{1},\ldots ,{\overset{↔}{X}}_{1}\;),$$

where ${\overset{↔}{X}}_{1}=\overset{↔}{Tr}\left(1,\;1,1,\;1\right)$ represents the lower linguistic value and ${\overset{↔}{X}}_{r}=\overset{↔}{Tr}\left(r,\;r,r,\;r\right)$ the higher linguistic value.

For the OS with r=5  levels, ${\overset{↔}{X}}_{1}=\overset{↔}{Tr}\left(1,\;1,1,\;1\right)$ represents linguistic value Very Bad, and ${\overset{↔}{X}}_{5}=\overset{↔}{Tr}\left(5,\;5,5,\;5\right)$—Very Good (see Table 2).

**Step 5.** For each alternative $A_{i}\;\left(i=1,2,\ldots ,m\right)$, calculate its distance from *PIS* using the following formula:

(28) $$d\left(A_{i},\;PIS\right)=\sum_{j=1}^{n}w_{j}\cdot d\left({\overset{↔}{X}}_{i,j},{\overset{↔}{X}}_{r}\;\right),$$

where $w_{j}$—weight of *j*th criterion (j=1,…,n), ${\overset{↔}{X}}_{i,j}$—evaluation alternative $A_{i}$ with respect to *j*th criterion,$d({\overset{↔}{X}}_{i,j},{\overset{↔}{X}}_{r}\;)$—the distance between trapezoidal oriented fuzzy numbers ${\overset{↔}{X}}_{i,j}$ and ${\overset{↔}{X}}_{r}\;$ is calculated using Formula (6).

**Step 6.** Calculate the synthetic measure for the ith alternative using one of the following formulas:

(29) $$OF-H1\left(A_{i}\right)=1-\frac{d\left(A_{i},\;PIS\right)}{d_{0}},$$

where $d_{0}=\overset{-}{d}+2S$, $\overset{-}{d}=\frac{1}{m}\sum_{i=1}^{m}d\left(A_{i},\;PIS\right)$, $S=\sqrt{\frac{1}{m}\sum_{i=1}^{m}{\left(d\left(A_{i},\;PIS\right)-\overset{-}{d}\right)}^{2}}$, or

(30) $$OF-H2\left(A_{i}\right)=1-\frac{d\left(A_{i},\;PIS\right)}{d\left(\;PIS,\;NIS\right)}=1-\frac{d\left(A_{i},\;PIS\right)}{2\left(r-1\right)},$$

where $d\left(PIS,\;NIS\right)=\sum_{j=1}^{n}w_{j}\cdot d({\overset{↔}{X}}_{r},{\overset{↔}{X}}_{1}\;)$ is the distance between *PIS* and *NIS*.

Let us observe that

(31) $$d\left(PIS,\;NIS\right)=\sum_{j=1}^{n}w_{j}\cdot d({\overset{↔}{X}}_{r},{\overset{↔}{X}}_{1}\;)=\sum_{j=1}^{n}w_{j}\cdot \sqrt{4{\left(r-1\right)}^{2}}=2{\left(r-1\right)}^{\;}.$$

**Step 7.** Rank all alternatives $A_{i}$ according to decreasing values $OF-H1\left(A_{i}\right)$ or $OF-H2\left(A_{i}\right)$.

Let us notice that in the classical Hellwig’s method, the ideal solution consists of the maximum values for the benefit criteria and minimum values for cost criteria, while the anti-ideal solution consists of the minimum values for benefit criteria and maximum values for cost criteria. In the proposed Hellwig’s approach based on OFNs, we identify the *NIS* as a solution consisting of the maximum values from the scale and *PIS* as a solution consisting of the minimum values from the scale. Therefore, there is no necessity of recalculating all results when a new alternative is introduced into consideration; consequently, this method avoids rank reversal. Because each offer is evaluated separately, we can omit the presentation of decision matrix, as in the classical Hellwig’s approach.

## 4. The Use of OF-Hellwig’s Approaches in Negotiation Support and Analysis

### 4.1. Prenegotiation Preparation and Determining the Scoring System

Although negotiation is stereotypically perceived as requiring the behavioral skills for effective communication and persuasion solely, the theory of negotiation clearly emphasizes that it is a complex process in which behavioral and formal perspectives on its conduct, analysis, and support interlace are equally important [1,79]. The formal approach is related to the economics embedded negotiation analysis [80,81]. It is focused on using a mathematical apparatus to help negotiators understand the negotiation problem better, conceptualize their preferences, and use them to support their decision regarding the selection of the negotiation contract that would mutually meet the aspirations of both parties in the best possible (and preferably fair) way. To this end, the negotiation analysis recommends pre-negotiation preparation during which, among other things, the parties jointly define the negotiation problem and formally specify it in the form of the negotiation template $T=\left\{I,{\left\{O_{j}\right\}}_{j=1,\ldots ,n}\right\}$, where $I=\left\{I_{1},I_{2},\ldots ,I_{n}\right\}$ is a set of n negotiation issues to be discussed during the negotiation, and $O_{j}$ are the sets of feasible negotiation options (resolution levels) for each negotiation issue j.

Having the negotiation template defined, the parties should individually declare the preferences (that reflect their goals and priorities) for all its elements, defining this way the scoring system S for the negotiation offers. Classically, such a scoring system is built assuming that the additive and fully compensatory model can adequately represent the negotiator’s preferences. The template is usually discrete (i.e., the sets $O_{j}$ are finite and limited to a few salient options only, $O_{j}=\left\{o_{j}^{1},o_{j}^{2}\ldots ,o_{j}^{n_{j}}\right\}$, when $n_{j}$ is the number of predefined salient options for jth issue), and the preferences are imparted quantitatively using a direct rating or point allocation mechanism (see, e.g., [80]). The negotiator declares the issue importance (weights) $w=\left(w_{1},w_{2},\ldots ,w_{n}\right)$ and the scores $v_{j}^{k}\;$ for each salient option of each issue from $O_{j}$ that comprise the set of options scores $V_{j}$ (for j=1,…,n). Thus, the scoring system is represented by the j+1-tuple $S=\left\{w,{\left\{V_{j}\right\}}_{j=1,\ldots ,n}\right\}$. The scoring system defined this way allows to evaluate any feasible negotiation offer by adding up the scores $v_{j}^{k}$ of the options that comprise this offer, i.e.,

(32) $$V\left(A_{i}\right)=\sum_{i=1}^{m}\sum_{j=1}^{n_{i}}z_{j}^{k}\left(A_{i}\right)\cdot v_{j}^{k},$$

where $z_{j}^{k}\left(A_{i}\right)$ is a binary variable indicating if the option $x_{j}^{k}$ comprises offer $A_{i}$ (1) or not (0).

From the technical viewpoint, scoring the negotiation template by an individual negotiator is equivalent to evaluating a multiple-criteria decision-making problem by a single decision maker. Template T defines all possible resolution levels the parties may negotiate for each issue while setting up a contract. Thus, it may be used to predefine the complete negotiation space N, which will specify all feasible offers (complete packages) that can be obtained as a list of all possible combinations of feasible options, one for each issue $I_{j}$. This space is formally defined as the Cartesian product of sets $O_{j}$, i.e.,

(33) $$N\left(T\right)=\prod_{j=1}^{n}O_{j}.$$

One can easily see that $N\left(T\right)$ is a typical set of alternatives that defines the decision matrix in a classic MCDM problem, as described in Section 3.2. Consequently, other scoring mechanisms could be applied, derived from the MCDM theory, to help negotiators declare their preferences more precisely but simultaneously in a less cognitively demanding way.

Finally, it is worth mentioning that template T is defined discretely through some selected salient options to make the definition of the problem and preferences more manageable for the parties, while the actual problem may be more complicated. Some of the issues might be, in fact, quantitative (and continuous), which would require defining the sets of their options through the feasible ranges (e.g., for the issue of price, it would be more convenient to declare that the feasible range is from 5.00 USD to 10.00 USD instead of defining each feasible price that differs in once cent from the others). However, a discrete template requires a lower workload while defining the negotiator preferences, as there are fewer scores to assign. If an offer that consists of the non-salient option needs to be analyzed later (during the bargaining phase), its score may be easily determined using the assumption of linear interpolation between the scores of two neighbouring salient options to the non-salient one. This is an equivalent of the assumption of pice-wise linear marginal scoring functions often used in other MCDM techniques, e.g., implicitly in UTA [82] or by introducing a bi-section scoring mechanism in SMARTS [83].

It is also worth mentioning that the further steps of prenegotiation preparation require the negotiators to define some reference alternatives called aspiration and reservation levels used as frames when evaluating the gains from the negotiation contract [1,84]. The aspiration level, usually conceptualized through an exemplary contract (complete package of options for all issues), defines the target value of the most wanted contract. While bargaining, the negotiators may always compare the actual offers to the aspiration one and figure out how far from their target they are. On the contrary, reservation level is often defined by BATNA, i.e., Best Alternative to Negotiation Agreement. BATNA refers to the solution that would be implemented when negotiation fails. In typical business negotiation, it is usually a contract that is attainable elsewhere (e.g., using the listed price contracting mechanism). BATNA should be evaluated using the scoring system developed beforehand, which results in defining the threshold of maximum concessions that the party may make in the negotiation. A rational negotiator should not accept an offer worse than BATNA, as it would result in losses.

With scoring systems, comprehensive support may be offered either individually or mutually to the parties. The former is provided when the classic iterative bargaining negotiation protocol is assumed, and parties do not make their preferences public. The latter is when the FOTE (full, open, truthful exchange) approach is used, and the parties jointly search for the best solution having complete information about their values to both sides. Scoring systems help parties evaluate each offer, measure the scale and reciprocity of concessions made by themselves and their counterparts, control the negotiation dynamics, analyze the efficiency of the negotiation agreement and search for its potential improvements. They may also be used by third parties to solve conflicts that reached deadlocks or breakdowns by implementing some arbitration mechanisms. A detailed description of both protocols and corresponding support mechanisms may be found in [80,81].

The potential use of the scoring systems for negotiation support that affect the negotiation process and outcomes clearly shows how important it is to ensure that they reflect the parties’ preferences adequately and truthfully. Hence, as mentioned earlier, the process of determining the scoring system should be straightforward, understandable, and easy to follow by the negotiator. The direct rating approach was considered easy; unfortunately, many negotiation experiments proved that the negotiators struggle with correctly interpreting the cardinal ratings and make many errors resulting from the use of heuristics and their cognitive biases [85,86]. Some other decision-making experiments also showed that negotiators with different information processing profiles might prefer not to define their preferences quantitatively but instead use some linguistic approaches. However, they may still expect some crisp numerical evaluations that would allow them to compare the offers and measure the scale of differences between them univocally [87,88]. Therefore, there is a constant need for designing a preference elicitation procedure that would be both easy to use and adequately represent the negotiator’s preferences. The OF-Hellwig’s approaches seem to be one of the potential candidates. They allow negotiators to operate with linguistic evaluations when defining preferences, conceptualize them using vague notions of the oriented fuzzy numbers and determine the quantitative global scores using commonly accepted notions of distances that do not require the negotiator to be involved in any cumbersome process of additional tuning the marginal scoring functions (as it may be required when some other additive models are in use). Therefore, we will examine their applicability to scoring the negotiation template and compare the results obtained this way to the ones obtained from similar techniques proposed earlier [17].

### 4.2. Determining Scoring Systems with OF-Hellwig’s Methods

We will consider a bilateral multi-issue purchase negotiation problem, typical in supply chain contracting (e.g., between a producer and its supplier). In the numerical example, the trapezoidal OFNs will be used to score a predefined template T and evaluate some examples of negotiation packages N. To make further comparisons of the results obtained with others presented in earlier studies [17] possible, we adopt a classic example of Cypress–Itex negotiations between bicycle produces (Cypress) and parts supplier (Itex), invented by InterNeg Research Center and implemented in many negotiation experiments conducted via the electronic negotiation system Inspire [5].

In our negotiations, the template consists of three following issues:

- $I_{1}$—unit price (in USD);
- $I_{2}$—returns conditions (described verbally by the fractions of accepted spoilage and percentage rate of the penalties);
- $I_{3}$—time of payment (in days).

Further, the sets of feasible options $O_{j}$ are defined within the template that comes from the initial verbal specification made by the parties, i.e.:

- For $I_{1}$, the unit price may vary from 20 USD to 42 USD, and the salient difference is considered to be 1 USD, hence
  $$O_{1}=\left\{20,\;21,22,\ldots ,42\right\};$$
- For $I_{2}$, return conditions are pre-defined through 13 feasible mixes of spoilage quota and penalty values, i.e.:
  $$O_{2}=\left\{\begin{array}{c} o_{2\;}^{1}=“5\%\;\mathrm{defects}\;\mathrm{and}\;4\%\;\mathrm{penalty}”;\;o_{2}^{2}=“6\%\;\mathrm{defects}\;\mathrm{and}\;4\%\;\mathrm{penalty}”;\\ o_{2}^{3}=“6.5\%\;\mathrm{defects}\;\mathrm{and}\;4\%\;\mathrm{penalty}”;\;o_{2}^{4}=“7\%\;defects\;and\;4\%\;penalty”,\;\\ o_{2}^{5}=“6\%\;\mathrm{defects}\;\mathrm{and}\;3\%\;\mathrm{penalty}”;o_{2}^{6}=“5\%\;\mathrm{defects}\;\mathrm{and}\;2\%\;\mathrm{penalty}”;\\ o_{2}^{7}=“5\%\;\mathrm{defects}\;\mathrm{and}\;1{.5\%\;\mathrm{penalty}”;\;o}_{2}^{8}=“3\%\;\mathrm{defects}\;\mathrm{and}\;1\%\;\mathrm{penalty}”;\\ o_{2}^{9}={“4\%\;\mathrm{defects}\;\mathrm{and}\;1\%\;\mathrm{penalty}”;\;o}_{2}^{10}=“3\%\;\mathrm{defects}\;\mathrm{and}\;\mathrm{no}\;\mathrm{penalty}”;\\ o_{2}^{11}=“3{.5\%\;\mathrm{defects}\;\mathrm{and}\;\mathrm{no}\;\mathrm{penalty}”;\;o}_{2}^{12}=“3.8\%\;\mathrm{defects}\;\mathrm{and}\;\mathrm{no}\;\mathrm{penalty}”;\\ o_{2}^{13}=“4\%\;\mathrm{defects}\;\mathrm{and}\;\mathrm{no}\;\mathrm{penalty}” \end{array}\right\}$$
- For $I_{3}$, time of payment varies from 1 to 24 days, hence
  $$O_{3}=\left\{1,\;2,3,\ldots ,24\right\}.$$

We will define the negotiation template and build the scoring systems for the seller in this negotiation, i.e., for the Itex party. For Itex, issue $I_{1}$ is a benefit criterion, and $I_{3}$ is a cost one.

Such a definition of the template is equivalent to the initial requirements for decision-making problem specification in Hellwig’s methods (requiring C and A to be explicitly specified, see Section 3.3). We may now follow the OF-Hellwig’s algorithm and score the negotiation template for Itex.

- **Step 1.**
  In the first step, Itex needs to define the order scale with the required numbers of levels and its quantitative equivalents defined by TrOFN-based *NOS*. We assume that Itex will use an extension of the 5-level linguistic scale with 13 linguistic evaluations and the corresponding *NOS*, as presented in Table 2 (Section 2.3).
- **Step 2.**
  We assume further that Itex defines the issue importance using any additional support procedure (developing of which is not the research interest of this study), resulting in the following vector of weights: $w=\left[0.6,\;0.2,\;0.2\right]$.
- **Step 3.**
  In the third step, the evaluation of all offers from A needs to be conducted using the scale defined in Step 1. Considering the specificity of negotiations, which are defined through the template, and the fact that it is often easier to evaluate the template than the entire negotiation space N directly, we recommend Itex focus on evaluating the template first.

Itex goes through the options defined in $O_{1},\;O_{2},$ and $O_{3}$ and evaluates them, which results in defining the scoring system S shown in Table 5.

The entire negotiation space N (an equivalent of A) that could be built based on the template T defined beforehand as a Cartesian product of all sets of options consists here of 23 × 13 × 24 = 7176 different packages. To make further analyses easier to follow, we will assume that Itex focuses on selected 15 negotiation packages only that, as it presumes, represent well the potential set of offers and counteroffers to be submitted by the parties during the forthcoming bargaining phase. These packages and single-issue linguistic evaluations of their component options are presented in Table 6.

Using the OFN-based *NOS* scale that accompanies the linguistics etiquettes (see Table 7), the decision (data) matrix may be built consisting of TrOFN evaluations of each offer. Such a matrix is shown in Table 7.

- **Step 4.**
  The *PIS* and *NIS* alternatives are now automatically defined using the ranges of the initial tentative OS scale applied, i.e., $PIS=\left(\overset{↔}{Tr}\left(1,1,1,1\right),\overset{↔}{Tr}\left(1,1,1,1\right),\overset{↔}{Tr}\left(1,1,1,1\right)\right)$ and $NIS=\left(\overset{↔}{Tr}\left(5,5,5,5\right),\overset{↔}{Tr}\left(5,5,5,5\right),\overset{↔}{Tr}\left(5,5,5,5\right)\right)$.
- **Steps 5 to 7.**
  The distances to *PIS* are determined for each alternative for A, and the synthetic measures that define the global scores of alternative OF-H1 and OF-H2 are calculated using Formulas (28)–(30).

To show some similarities of the results obtained to those that may be delivered by another reference point-based method, i.e., TOPSIS, we have also calculated the TOPSIS-based ratings for the 15 alternatives from Table 7 using *r*-levels (*r* = 5) linguistic scale. The Oriented Fuzzy TOPSIS [17,22] differs from Hellwig’s approaches in using the distance to *NIS* for calculating the global rating (separation measure), which is performed in the following way:

(34) $$OF-T\left(A_{i}\right)=\frac{d\left(A_{i},\;NIS\right)}{d\left(A_{i},\;NIS\right)+d\left(A_{i},\;PIS\right)}=\frac{\sum_{j=1}^{n}w_{j}\cdot d\left({\overset{↔}{X}}_{i,j},{\overset{↔}{X}}_{1}\;\right)}{\sum_{j=1}^{n}w_{j}\cdot d\left({\overset{↔}{X}}_{i,j},{\overset{↔}{X}}_{1}\;\right)+\sum_{j=1}^{n}w_{j}\cdot d\left({\overset{↔}{X}}_{i,j},{\overset{↔}{X}}_{r}\;\right)},$$

where $w_{j}$—weight of *j*th criterion (j=1,…,n), ${\overset{↔}{X}}_{i,j}\;$—evaluation of alternative $A_{i}\;$ with respect to *j*th criterion,$\;d({\overset{↔}{X}}_{i,j},{\overset{↔}{X}}_{1}\;)$, $d({\overset{↔}{X}}_{i,j},{\overset{↔}{X}}_{r}\;)$—distances between trapezoidal oriented fuzzy numbers ${\overset{↔}{X}}_{i,j}$ and $\;{\overset{↔}{X}}_{1}\;(\;{\overset{↔}{X}}_{r})$ is calculated using Formula (6).

The negotiation packages’ ratings and rankings determined by OF-TOPSIS and OF-Hellwig’s variants are presented in Table 8.

To gain a broader perspective on how such reference-points-based ratings and rankings may differ from other results obtained when OFNs are used in the simplest additive aggregation, we finally computed the rating according to OF-SAW [17,21] technique. The latter defines an oriented fuzzy global score of an alternative which is determined in the following way:

(35) $$OF-S\left(A_{i}\right)=w_{1}⊡{\overset{↔}{X}}_{i,1}⊞w_{2}⊡{\overset{↔}{X}}_{i,2}⊞\ldots ⊞w_{n}⊡{\overset{↔}{X}}_{i,n},$$

where $w_{j}$—weight of *j*th criterion, ${\overset{↔}{X}}_{i,j}$—evaluation of alternative $A_{i}\;$ with respect to *j*th criterion (j=1,…,n).

Let as recall that symbol ⊞ means addition of oriented fuzzy numbers (see Formula (3)), while ⊡—multiplication real number by oriented fuzzy number (see Formula (4)).

As the Oriented Fuzzy SAW method determines aggregated evaluation coefficients in the form of TrOFN, the defuzzification formulas are required to obtain crisp evaluations of offers and resulting rank ordering. The following formulas may be applied (see Table 1).

The values of the SAW measures, along with crisp evaluation resulting from different defuzzification formulas and the ranks of packages, are presented in Table 9.

## 5. Discussion

Let us first note that all techniques considered in Section 4, i.e., OF-SAW, OF-TOPSIS, and both OF-Hellwig’s methods, allow for assigning a quantitative score to each offer and ordering them from best to worst. It is a desired property of the negotiation offer scoring system because we can estimate the value of each offer and counter offer and evaluate the value of the concessions made. What is also important is that all considered methods are based on a verbal evaluation of options using the linguistic scale. It allows us to implement them in the same negotiation situation, in which parties cannot (e.g., due to some cognitive limitations) classically evaluate the template employing crisp values assigned, for instance, through the direct rating approach. Consequently, Steps 1–3, which are used in both OF-Hellwig’s methods to score the negotiation template (and described in Section 3.3), are also performed in the same way in OF-SAW and OF-TOPSIS.

Similarly, all the methods require defining the issue importance in the form of a vector of weights (as shown in Step 4 for the OF-Hellwig’s procedure). As a result, they all use the same formally defined scoring system. What makes the methods different is the aggregation procedure used to produce the global scores of alternatives. Below, we discuss the main methodological differences in algorithms. In particular, we pay attention to similarities and dissimilarities among methods and their advantages and disadvantages when applied to support building a negotiation scoring system.

Let us observe that linguistic evaluation of negotiation options eliminates the problem of differentiation between benefit and cost criteria and the necessity of criteria normalization (see Step 4, Formulas (18) and (19)). The OF-T and OF-H2 measures are normalized in the range [0, 1], but OF-H1 is not. The latter allows some offers to be evaluated below 0. These are the worst offers in the negotiation space, the performance of which is so bad that they occur more distant from *PIS* than those with a distance equal to average plus two values of standard deviation.

The OF-T and OF-H2 measure require building both an ideal solution (*PIS*) and an anti-ideal (*NIS*) solution, while OF-H1requires only an ideal solution. The OF-S measure does not require such reference points. Those reference points can be considered aspiration (*PIS*) and reservation (*NIS*) levels in the negotiation analysis. We identify the *PIS* as a solution consisting of the maximum values from the scale. Simultaneously, we identify the *NIS* as a solution consisting of the minimum values from the scale. Let us recall that the OF-TOPSIS method is based on the idea that the chosen alternative should be the closest to the positive ideal solution and the farthest from the negative ideal solution. Yet, in OF-Hellwig-based techniques, the chosen alternative should be the closest to the positive ideal solution, although in OF-H2 the distances between the best (positive ideal) and the worst (negative ideal) solutions were used to normalize the measure. It is challenging to univocally confirm which use of reference points results in the definition of the global scores more precisely and adequately reflecting the negotiator’s preferences. It seems it should be a DM’s individual decision based on prior training in using all these methods that would show the hands-on results of each approach using some numerical examples.

The advantage of constructing *PIS* and *NIS* using TrOFNs corresponding to the minimum and maximum levels from the linguistic scale is that if a new offer is included or one of the existing ones is removed from N, there is no need to reevaluate the offers previously scored through OF-T, OF-H2 and OF-S techniques. In addition, scores of all alternatives remain stable. Thus, those techniques avoid rank reversals. Unfortunately, it does not hold for the OF-H1 technique, which is normalized by aggregated scores of alternatives. Therefore, adding or removing alternatives provides a stable ranking, but the scores of alternatives can be changed. A good example of how significant these changes can be is shown in Table 10 below, where the global scores of 15 alternatives obtained through OF-H1 are shown, first determined based on the limited negotiation space $N_{1}$ (consisting of 15 alternatives) and then based on the entire space of feasible alternatives $N_{2}$ (consisting of 7176) built out of the template presented in Table 5.

Such differences are the result of the peculiarity of the scoring formula, which is sensitive to the number and performance of all alternatives *m* that are processed to determine the average distance, standard deviation across the negotiation space and $d_{0}$ value according to Formula (28). Even if differences may not be significant for some offers, as for P15, where the discrepancies reach only 0.147−0.044=0.034 rating points, for others, they may reach as much as 0.1 rating points, e.g., for P1 and P9. It means nearly 10% of the entire rating space. Such a situation does not seem comfortable from the perspective of negotiation support, as the negotiator receives an ambiguous recommendation on the potential good quality of the same offers and is left confused about which scores should be considered a reliable basis when analyzing the potential concessions.

It is also worth noting that even though OF-H1 does not use the second reference point (*NIS*) explicitly in preference declarations, it is implicitly used to determine the global score. An equivalent of *NIS* is considered an offer, the distance of which to *PIS* equals an average value of all distances in negotiation space N plus two values of their standard deviation. It is used to set a reference threshold of a global score equal to 0. Hence, despite the global values in OF-H1 not being limited to [0; 1]-range as they are in OF-T and OF-H2, those that fit this range may be considered as varying between two reference points: the best and the “very bad” one. OF-H1 additionally allows considering some offers to be “worse than very bad” and scoring them with negative values if they are worse than the one implicitly defined as *NIS* (with a score equal to 0).

The discussion above makes us propose an additional minor modification to the OF-H1-based Hellwig’s method to determine the global scores that would better fit the negotiator’s individual problem understanding. Instead of using an implicit reference point that refers to what statistics consider an outlier but which may be too abstract for any negotiation party, the negotiator may clearly define what their BATNA is pointing out to one (or more) borderline offers, i.e., the one (ones) that embody maximum concessions they are going to offer to their counterpart. Then, in Formula (28), the denominator in the subtracting fraction $d_{0}$ should be determined as

(36) $$d_{0}=\frac{1}{m_{B}}\sum_{i=1}^{m_{B}}d\left(A_{i}^{B},\;PIS\right),$$

where: $A_{i}^{B}$ is the set of alternatives defined by the negotiator as their BATNA offers, and $m_{B}=\left|A_{i}^{B}\right|$.

Another issue should also be considered when comparing OF-Hellwig’s approaches to the OF-SAW one. The latter is based on a weighted average of performance ratings and results in the global scores represented by oriented fuzzy numbers. To compare the offers and determine the scale of concessions between them, the negotiator has to use one of the methods of defuzzification (see Table 1). However, various defuzzification approaches process imprecise information differently, resulting in different evaluations of alternatives and offer rankings. Again, the selection of an “appropriate” defuzzification approach requires additional cognitive effort from the decision maker to understand how these approaches work and what are the numerical consequences of selecting each of them.

**Figure 4:**
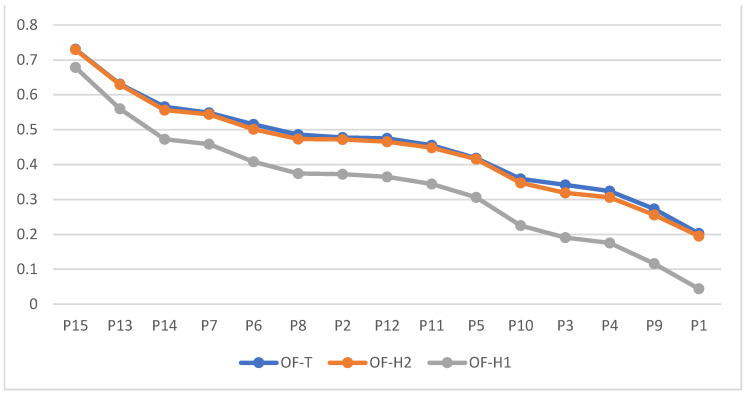
Global scores of 15 packages considered by Itex for OF-T, OF-H1, and OF-H2 scoring functions.

Analyzing the differences between the methods using the example shown in Section 4.2 also provides some interesting insights from the viewpoint of negotiation analysis and support. The results obtained from two OF-Hellwig’s methods and OF-TOPSIS and OF-SAW may, at first glance, look similar. However, more detailed analyses reveal the differences that may have a crucial impact on further use of the global ratings obtained from each method in the forthcoming actual negotiations phase. First, if both Hellwig’s methods and OF-TOPSIS are compared, one may easily notice that all three produce the same offers rankings. Apart from the Kendall correlation coefficient, which is naturally equal to 1 for all pairs of ranking considered, the Person correlations among the series of offers ratings are also exceptionally high (>0.999). The data series visualizing the differences in rating for all three methods and the 15 alternatives evaluated with the negotiation space $N_{1}$ are shown in Figure 4.

**Figure 5:**
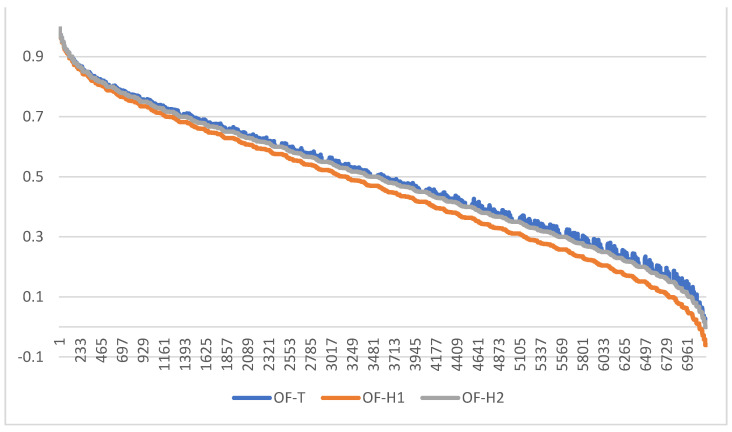
The systemic differences in offers evaluation for all offers from the negotiation space $N_{2}$ and considered by Itex and OF-T, OF-H1, and OF-H2 scoring functions.

From the viewpoint of developing the concession strategy in prenegotiation [1], all three methods indicate the same sequence of offers to make the subsequent minimal concessions. However, the conclusions vary if we analyze how Itex may interpret the relative value of subsequent offers and the scale of concessions related to submitting the successive packages from the list. OF-T and OF-H2 measures generate nearly the same offer ratings and assure that Itex will interpret the quality of offers likewise, no matter which scoring aggregation mechanism is used. For instance, P15 is evaluated equally as fairly good by these methods, i.e., $OF-T\left(P15\right)\;$ = $OF-H2\left(P15\right)$ = 0.73. The next offer is also evaluated the same by both methods, i.e., $OF-T\left(P13\right)$ = $OF-H2\left(P13\right)$ = 0.63. Consequently, the concession is considered worth 0.1 rating points, no matter which method is used. However, if OF-H1 is applied, the relative interpretation of offers value and concessions differ, i.e., $OF-H1\left(P15\right)$ = 0.68, which is 0.05 rating points worse than when evaluated by OF-T or OF-H2. One may consider such difference to be minor, but note that the rating space in the OF-TOPSIS and OF-Hellwig’s *H*2 methods is scaled to the [0; 1]-range (and for *H*1, this range still allows for considering the distance between the very best and the significantly bad offer); thus, the difference of 0.05 means (at least) five percentage points. The difference in interpreting offers quality increases more and more for subsequent offers in the order depending on whether OF-H1-based ratings or those resulting from OF-T or OF-H2 are used (the gap between blue/orange line and the grey one increases while moving to the right). The least attractive offer P1 is scored 0.2 by OF-T and OF-H2-based rating formulas, but only 0.04 according to OF-H1. The discrepancy is significant. Similarly, the interpretation of the scale of concessions may differ heavily for those methods. For instance, the first concession, made when Itex resigns from P15 and submits to the negotiation table P13 instead, is worth $OF-T\left(P15\right)-OF-T\left(P13\right)$ = $OF-H2\left(P15\right)-OF-H2\left(13\right)$ = 0.10 but as much as 0.12 points when OF-H1-based scoring system is used. Even minor differences in measuring the amounts of concessions may impact negotiators’ attitudes toward the counterpart and perception of reciprocity. Therefore, it is crucial which of these methods will be implemented to support negotiators, and the meanings of the scores and scale of scoring space should be thoroughly explained to them to avoid future misinterpretations of negotiation moves. All these discrepancies in the evaluation of the offers discussed above have a systemic character and influence all the offers within the entire negotiation space defined for this negotiation problem by the template T, i.e., when the negotiation space $N_{2}$ consisting of 7176 offers is evaluated (see Figure 5).

What also should be noticed is that OF-H1 and OF-H2 generate ratings that produce more similar ranks of offers than those obtained from OF-T. It can be easily seen from Figure 5 that OF-H1 and OF-H2 consequently decrease for subsequent negotiation offers (when moving from left to right of the chart) in regular and symmetric deviations. This cannot be observed for OF-T, which, despite generating ratings similar in values to those from OF-H2, results in ranks that frequently differ. It can be observed through the regular picks of the blue data series when confronted with the smooth decrease of the grey one.

**Figure 6:**
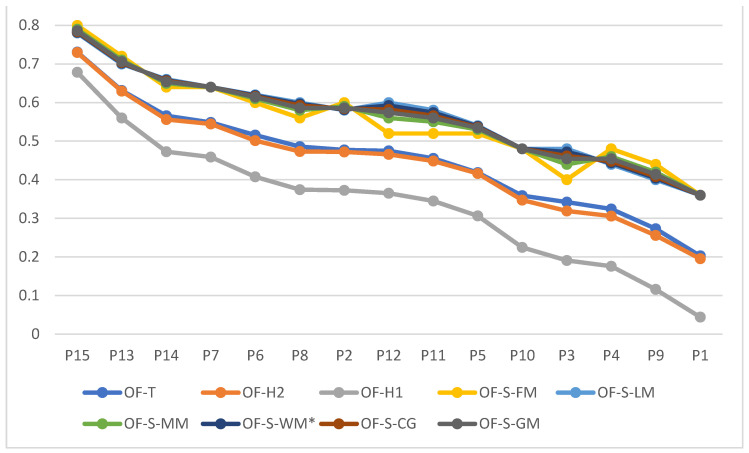
Global scores of 15 packages considered by Itex for OF-S, OF-T, OF-H1, and OF-H2 scoring functions.

Given the above, the comparison of results obtained from OFN-based reference-points-based approaches (i.e., TOPSIS and Hellwig) with those coming from OF-SAW and various defuzzification techniques seems interesting. To show the similarities in the evaluations on a common graph, we normalized the OF-SAW ratings from Table 9 using max–min normalization with scores 1 and 5 as min and max, respectively. The global scores of 15 offers are shown in Figure 6.

Generally, we may observe that OF-SAW-based global ratings indicate that offers are more attractive to Itex. Their relative scores are higher (after scaling them) than the corresponding ones obtained from OF-TOPSIS or OF-Hellwig approaches. The differences in offers evaluations, important from the viewpoint of measuring the scale of the concessions, are even smaller for OF-S measure than for OF-T or - OF-H2 (the data series are far more flat). As a result, interpreting the value of the worst offer considered by Itex may bring different conclusions, depending on the approach used. Offer P1 evaluated by any OF-SAW techniques will be considered somewhat weak (with a rating of about 0.36 points), while from the viewpoint of OF-H1-based rating it would be totally unacceptable (with a rating of 0.04). Again, the discrepancy in evaluating the best offer (P15) will not be so evident.

## 6. Conclusions

In this paper, we proposed two variants of the OF-Hellwig’s approaches as extensions of the classical Hellwig’s method and analyzed the usability of those techniques in negotiation support.

The contribution of this paper is as follows. Firstly, we proposed two variants of Hellwig-based methods based on oriented fuzzy numbers and a linguistic approach. The oriented fuzzy numbers are used to deal with imprecise and incomplete information in the negotiation process. In the first classical variant (OF-H1), we used Hellwig’s proposition of normalization measure based on the average and the standard deviation of distances between alternatives and the ideal solution. In the second variant (OF-H2), we used a normalized measure based on the distance between ideal and anti-ideal solutions represented by a linguistic scale which makes the OF-Hellwig’s algorithm more straightforward and intuitive than the classical approach.

Secondly, we showed how these fuzzy Hellwig-based techniques might be applied for building a negotiation scoring system in multi-issue negotiations and analyzed their usability in the illustrative example. Finally, the proposed methods were compared with other techniques proposed earlier in the literature, i.e., SAW and TOPSIS, based on the oriented fuzzy numbers. In this comparison, we considered both the technical properties of algorithms and the results from the illustrative example. The advantages and disadvantages of all techniques seen from the viewpoint of the negotiation support focused on building a scoring system were also outlined.

The comparative analysis showed that the OF-H1 and OF-H2 measures might be an alternative for other techniques based on oriented fuzzy numbers such as OF-S or OF-T. The main advantage of OF-Hellwig’s techniques compared to OF-SAW is that it does not use defuzzification formulas. In contrast, compared to OF-TOPSIS, it may be based on one reference point (ideal solution, as in H1) or adopt the second reference point to the individual needs of the negotiators (additional definition of BATNA used to normalize the measure). Finally, comparing OF-H1 and OF-H2, the latter seems simpler and more intuitive and additionally avoids changing scores when a set of offers is modified. However, we should note that DM has to decide which of the methods will be the most useful in a specific negotiation situation and which of them best reflects preferences through the global score points. Let us recall that in our example, OF-T and OF-H2 produced highly similar scores of offers, though they may result in different rankings. At the same time, OF-H1 and OF-H2 algorithms produce highly similar rankings but with quite high differences in scoring points. Consequently, using different methods will impact the interpretation of not only the offers’ scores, but also the entire negotiation process, i.e., the concession made by both parties, the negotiation progress and the agreement. Additionally, not discussed broader in this paper, it will have a significant impact on the recommendation of compromise improvement in the post-negotiation phase, as well as the results of the potential negotiation support offered by the third party (e.g., the suggestions of fair agreements made by the arbitrator).

Our future research will be focused on empirically verifying the negotiators’ acceptability of the OF-Hellwig’s methods. The negotiation experiments conducted with the support of OF-H1 and OF-H2  measures could allow us to verify if the reception of these negotiation support mechanisms is positive and whether it may depend on the cognitive profiles of the parties. Additionally, the applicability of these approaches may also be verified in decision-making contexts other than negotiations.

## Figures and Tables

**Table 1:** Methods of defuzzification.

Functional	Formula
weighted maximum functional (WM)	$\phi_{WM}\left(\overset{↔}{Tr}\left(a,b,c,d\right)|\lambda \right)=\lambda \cdot b+\left(1-\lambda \right)\cdot c,\;\;\lambda \in \left[0;1\right]$
first maximum functional (FM)	$\phi_{FM}\left(\overset{↔}{Tr}\left(a,b,c,d\right)\right)=\phi_{WM}\left(\overset{↔}{Tr}\left(a,b,c,d\right)|1\right)=b$
last maximum functional (LM)	$\phi_{LM}\left(\overset{↔}{Tr}\left(a,b,c,d\right)\right)=\phi_{WM}\left(\overset{↔}{Tr}\left(a,b,c,d\right)|0\right)=c$
middle maximum functional (MM)	$\phi_{MM}\left(\overset{↔}{Tr}\left(a,b,c,d\right)\right)=\phi_{WM}\left(\overset{↔}{Tr}\left(a,b,c,d\right)|\frac{1}{2}\right)=\frac{1}{2}\cdot \left(b+c\right)$
gravity center functional (GC)	$\phi_{CG}\left(\overset{↔}{Tr}\left(a,b,c,d\right)\right)=\left\{\begin{array}{c} \frac{a^{2}+a\cdot b+b^{2}-c^{2}-c\cdot d-d^{2}}{3\left(a+b-c-d\right)},\;\;\;\;\;\;\;\;\;a\neq d\\ \;a,\;\;\;\;\;\;\;\;\;\;\;\;\;\;\;\;\;\;\;\;\;\;\;\;\;\;\;\;\;\;\;\;\;\;\;\;\;\;\;\;\;\;\;\;a=d \end{array}\right.$
geometrical mean functional (GM)	$\phi_{GM}\left(\overset{↔}{Tr}\left(a,b,c,d\right)\right)=\left\{\begin{array}{c} \frac{a\cdot b-c\cdot d}{a+b-c-d},\;\;\;\;\;\;\;\;\;\;a\neq d\\ \;a\;\;\;\;\;\;\;\;\;\;\;\;\;\;\;\;\;\;\;\;\;\;\;a=d \end{array}\right.$

Source: own.

**Table 2:** Transformation of linguistic values into Numerical Order Scale NOS.

Linguistic Values	Order Scale	Numerical Diagnosis Set	Numerical Order Scale NOS
Very Bad	VB	1	$\overset{↔}{Tr}\left(1,1,1,1\right)$
at least Very Bad	L.VB	GE.1	$\overset{↔}{Tr}\left(1,1,1.5,2\right)$
at most Bad	M.B	LE.2	$\overset{↔}{Tr}\left(2,2,1.5,1\right)$
Bad	B	2	$\overset{↔}{Tr}\left(2,2,2,2\right)$
at least Bad	L.B	GE.2	$\overset{↔}{Tr}\left(2,2,2.5,3\right)$
at most average	M.AV	LE.3	$\overset{↔}{Tr}\left(3,3,2.5,2\right)$
Average	AV	3	$\overset{↔}{Tr}\left(3,3,3,3\right)$
at least average	L.AV	GE.3	$\overset{↔}{Tr}\left(3,3,3.5,4\right)$
at most good	M.G	LE.4	$\overset{↔}{Tr}\left(4,4,3.5,3\right)$
Good	G	4	$\overset{↔}{Tr}\left(4,4,4,4\right)$
at least good	L.G	GE.4	$\overset{↔}{Tr}\left(4,4,4.5,5\right)$
at most Very Good	M.VG	LE.5	$\overset{↔}{Tr}\left(5,5,4.5,4\right)$
Very Good	VG	5	$\overset{↔}{Tr}\left(5,5,5,5\right)$

Source: Own.

**Table 3:** Applications and modifications of classical Hellwig’s procedure (1968).

Authors	Type of Procedure	Application
Hellwig (1972) [50]	original approach	evaluation of socio-economic development for different countries
Łuczak and Wysocki (2007) [53]	modified (fuzzy synthetic measure)	evaluation of the socio-economic development of rural Wielkopolska
Di Domizio (2008) [57]	original approach	study of the competitive balance of the Italian Football League
Wysocki (2010) [58]	original approach	recognizing economic types of agriculture and rural areas
Pawlas (2016) [59]	original approach	analysis disparities in the economic development of 28 EU member states in 2014
Reiff et al. (2016) [60]	original approach	analysis differences in agriculture performance across the European Union countries in the years 2010–2013
Gałecka and Smolny (2018) [61]	original approach	evaluation of theater activity in Poland
Iwacewicz-Orłowska and Sokołowska (2018) [62]	original approach	analysis of the indicators of sustainable development concerning environmental governance
Krukowski et al. (2018) [63]	original approach	evaluation of agriculture development in the member states of the European Union in the years 2007–2015
Jefmański (2019) [54]	modified (intuitionistic fuzzy synthetic measure for ordinal data)	evaluation of the quality-of-life research of the residents of the communes of the Kraina Łęgów Odrzańskich region in Poland
Roszkowska (2021) [55]	modified (intuitionistic fuzzy ideal reference point approach)	evaluation of negotiation offers
Roszkowska and Filipowicz-Chomko (2021) [52]	modified (an approach based on individual patterns)	evaluation of the implementation of the Europe 2020 strategy in education across EU countries
Roszkowska and Jefmański (2021) [56]	modified (interval-valued intuitionistic fuzzy synthetic measure)	analysis of survey data
Kusterka-Jefmańska et al. [64]	modified (intuitionistic fuzzy synthetic measure)	analysis of subjective quality of life in EU cities based on survey data

Source: Own.

**Table 4:** Modifications and applications of the second variant of the classical Hellwig’s procedure (1981).

Authors	Type of Procedure	Application
Walesiak and Dehnel (2019, 2020) [65,66]	modified (synthetic measure for interval-valued symbolic data)	evaluation of economic efficiency of medium-sized manufacturing enterprises in districts of Wielkopolska province; assessment of social cohesion in provinces of Poland in 2018
Dehnel et al. (2020) [67]	modified (applying multidimensional scaling)	comparison of the variation in population aging in four Visegrad countries (V4) and across their NUTS2 regions in 2016 and 2005
Roszkowska et al. (2022) [68]	modified (the double intuitionistic fuzzy synthetic measure)	choice of air-conditioning system installed in a library

Source: own.

**Table 5:** Negotiation template with its scoring system defined by Itex through the OFN-based scale.

Unit Price	Order Scale	Returns Conditions	Order Scale	Time of Payment	Order Scale
20 to 22	VB	5% defects and 4% penalty	VB	21 to 24	VB
23	L.VB	6% defects and 4% penalty	L.VB	20	L.VB
24	M.B	6.5% defects and 4% penalty	M.B	19	M.B
25 to 27	B	7% defects and 4% penalty	B	16 to 18	B
28	L.B	6% defects and 3% penalty	L.B	15	L.B
29	M.AV	5% defects and 2% penalty	M.AV	14	M.AV
30 to 32	AV	5% defects and 1.5% penalty	AV	11 to 13	AV
33	L.AV	3% defects and 1% penalty	L.AV	10	L.AV
34	M.G	4% defects and 1% penalty	M.G	9	M.G
35 to 37	G	3% defects and no penalty	G	6 to 8	G
38	L.G	3.5% defects and no penalty	L.G	5	L.G
39	M.VG	3.8% defects and no penalty	M.VG	4	M.VG
40 to 42	VG	4% defects and no penalty	VG	1 to 3	VG

Source: own.

**Table 6:** Negotiations packages and their linguistic evaluation.

Package	Unit Price		Returns Conditions		Time of Payment	
	Option	Linguistic Evaluation	Option	Linguistic Evaluation	Option	Linguistic Evaluation
P1	21	VB	6% defects and 3% penalty	L.B	9	M.G
P2	27	B	3.8% defects and no penalty	M.VG	7	G
P3	23	L.VB	3% defects and no penalty	G	10	L.AV
P4	24	M.B	5% defects and 1.5% penalty	AV	10	L.AV
P5	25	B	3% defects and 1% penalty	L.AV	8	G
P6	28	L.B	4% defects and 1% penalty	M.G	4	M.VG
P7	32	AV	4% defects and 1% penalty	M.G	10	L.AV
P8	28	L.B	5% defects and 1.5% penalty	AV	4	M.VG
P9	29	M.AV	6% defects and 4% penalty	L.VB	23	VB
P10	31	AV	6.5% defects and 4% penalty	M.B	20	L.VB
P11	33	L.AV	7% defects and 4% penalty	B	18	B
P12	33	L.AV	6% defects and 3% penalty	L.B	17	B
P13	37	G	5% defects and 1.5% penalty	AV	14	M.AV
P14	33	L.AV	4% defects and 1% penalty	M.G	14	M.AV
P15	41	VG	7% defects and 4% penalty	B	14	M.AV

Source: [17].

**Table 7:** Decision matrix consisting of 15 negotiation packages defined by Itex.

Package	Unit Price	Returns Conditions	Time of Payment
P1	$\overset{↔}{Tr}\left(1,1,1,1\right)$	$\overset{↔}{Tr}\left(2,2,2.5,3\right)$	$\overset{↔}{Tr}\left(4,4,3.5,3\right)$
P2	$\overset{↔}{Tr}\left(2,2,2,2\right)$	$\overset{↔}{Tr}\left(5,5,4.5,4\right)$	$\overset{↔}{Tr}\left(4,4,4,4\right)$
P3	$\overset{↔}{Tr}\left(1,1,1.5,2\right)$	$\overset{↔}{Tr}\left(4,4,4,4\right)$	$\overset{↔}{Tr}\left(3,3,3.5,4\right)$
P4	$\overset{↔}{Tr}\left(2,2,1.5,1\right)$	$\overset{↔}{Tr}\left(3,3,3,3\right)$	$\overset{↔}{Tr}\left(3,3,3.5,4\right)$
P5	$\overset{↔}{Tr}\left(2,2,2,2\right)$	$\overset{↔}{Tr}\left(3,3,3.5,4\right)$	$\overset{↔}{Tr}\left(4,4,4,4\right)$
P6	$\overset{↔}{Tr}\left(2,2,2.5,3\right)$	$\overset{↔}{Tr}\left(4,4,3.5,3\right)$	$\overset{↔}{Tr}\left(5,5,4.5,4\right)$
P7	$\overset{↔}{Tr}\left(3,3,3,3\right)$	$\overset{↔}{Tr}\left(4,4,3.5,3\right)$	$\overset{↔}{Tr}\left(3,3,3.5,4\right)$
P8	$\overset{↔}{Tr}\left(2,2,2.5,3\right)$	$\overset{↔}{Tr}\left(3,3,3,3\right)$	$\overset{↔}{Tr}\left(5,5,4.5,4\right)$
P9	$\overset{↔}{Tr}\left(3,3,2.5,2\right)$	$\overset{↔}{Tr}\left(1,1,1.5,2\right)$	$\overset{↔}{Tr}\left(1,1,1,1\right)$
P10	$\overset{↔}{Tr}\left(3,3,3,3\right)$	$\overset{↔}{Tr}\left(2,2,1.5,1\right)$	$\overset{↔}{Tr}\left(1,1,1.5,2\right)$
P11	$\overset{↔}{Tr}\left(3,3,3.5,4\right)$	$\overset{↔}{Tr}\left(2,2,2,2\right)$	$\overset{↔}{Tr}\left(2,2,2,2\right)$
P12	$\overset{↔}{Tr}\left(3,3,3.5,4\right)$	$\overset{↔}{Tr}\left(2,2,2.5,3\right)$	$\overset{↔}{Tr}\left(2,2,2,2\right)$
P13	$\overset{↔}{Tr}\left(4,4,4,4\right)$	$\overset{↔}{Tr}\left(3,3,3,3\right)$	$\overset{↔}{Tr}\left(3,3,2.5,2\right)$
P14	$\overset{↔}{Tr}\left(3,3,3.5,4\right)$	$\overset{↔}{Tr}\left(4,4,3.5,3\right)$	$\overset{↔}{Tr}\left(3,3,2.5,2\right)$
P15	$\overset{↔}{Tr}\left(5,5,5,5\right)$	$\overset{↔}{Tr}\left(2,2,2,2\right)$	$\overset{↔}{Tr}\left(3,3,2.5,2\right)$

Source: [17].

**Table 8:** The negotiation packages’ rankings determined by OF-TOPSIS and OF-Hellwig’s methods.

Packages	$d\left(A_{i},\;PIS\right)$	$d\left(A_{i},\;NIS\right)$	OF-TOPSIS		OF-Hellwig’s			
			OF-T	Rank	OF-H1	Rank	OF-H2	Rank
P1	6.437	1.637	0.203	15	0.044	15	0.195	15
P2	4.224	3.859	0.477	7	0.373	7	0.472	7
P3	5.449	2.835	0.342	12	0.191	12	0.319	12
P4	5.551	2.664	0.324	13	0.176	13	0.306	13
P5	4.671	3.364	0.419	10	0.306	10	0.416	10
P6	3.987	4.246	0.516	5	0.408	5	0.502	5
P7	3.645	4.427	0.548	4	0.459	4	0.544	4
P8	4.213	3.983	0.486	6	0.374	6	0.473	6
P9	5.953	2.236	0.273	14	0.116	14	0.256	14
P10	5.220	2.924	0.359	11	0.225	11	0.348	11
P11	4.412	3.693	0.456	9	0.345	9	0.448	9
P12	4.275	3.868	0.475	8	0.365	8	0.466	8
P13	2.964	5.071	0.631	2	0.560	2	0.629	2
P14	3.551	4.627	0.566	3	0.473	3	0.556	3
P15	2.164	5.871	0.731	1	0.679	1	0.729	1

Source: Own.

**Table 9:** Package evaluations obtained by OF-SAW methods and using defuzzification.

Package	$OF-S\left(A_{i}\right)$	Deffuzied OF-S (OF-S-d)						RANK					
		FM	LM	MM	WM *	CG	GM	FM	LM	MM	WM *	CG	GM
P1	$\overset{↔}{Tr}\left(1.8,1.8,1.8,1.8\right)$	1.80	1.80	1.80	1.80	1.80	1.80	15	15	15	15	15	15
P2	$\overset{↔}{Tr}\left(3.0,3.0,2.9,2.8\right)$	3.00	2.90	2.95	2.91	2.92	2.93	5.5	8.5	6	8	7	6.5
P3	$\overset{↔}{Tr}\left(2.0,2.0,2.4,2.8\right)$	2.00	2.40	2.20	2.36	2.31	2.27	14	11.5	13	12	12	12.5
P4	$\overset{↔}{Tr}\left(2.4,2.4,2.2,2.0\right)$	2.40	2.20	2.30	2.22	2.24	2.27	11.5	13	12	13	13	12.5
P5	$\overset{↔}{Tr}\left(2.6,2.6,2.7,2.8\right)$	2.60	2.70	2.65	2.69	2.68	2.67	9	10	10	10	10	10
P6	$\overset{↔}{Tr}\left(3.0,3.0,3.1,3.2\right)$	3.00	3.10	3.05	3.09	3.08	3.07	5.5	5	5	5	5	5
P7	$\overset{↔}{Tr}\left(3.2,3.2,3.2,3.2\right)$	3.20	3.20	3.20	3.20	3.20	3.20	3.5	4	4	4	4	4
P8	$\overset{↔}{Tr}\left(2.8,2.8,3.0,3.2\right)$	2.80	3.00	2.90	2.98	2.96	2.93	7	6.5	7	6	6	6.5
P9	$\overset{↔}{Tr}\left(2.2,2.2,2.0,1.8\right)$	2.20	2.00	2.10	2.02	2.04	2.07	13	14	14	14	14	14
P10	$\overset{↔}{Tr}\left(2.4,2.4,2.4,2.4\right)$	2.40	2.40	2.40	2.40	2.40	2.40	11.5	11.5	11	11	11	11
P11	$\overset{↔}{Tr}\left(2.6,2.6,2.9,3.2\right)$	2.60	2.90	2.75	2.87	2.83	2.80	9	8.5	9	9	9	9
P12	$\overset{↔}{Tr}\left(2.6,2.6,3.0,3.4\right)$	2.60	3.00	2.80	2.96	2.91	2.87	9	6.5	8	7	8	8
P13	$\overset{↔}{Tr}\left(3.6,3.6,3.5,3.4\right)$	3.60	3.50	3.55	3.51	3.52	3.53	2	2	2	2	2	2
P14	$\overset{↔}{Tr}\left(3.2,3.2,3.3,3.4\right)$	3.20	3.30	3.25	3.29	3.28	3.27	3.5	3	3	3	3	3
P15	$\overset{↔}{Tr}\left(4.0,4.0,3.9,3.8\right)$	4.00	3.90	3.95	3.91	3.92	3.93	1	1	1	1	1	1

Source: [17] *—λ=0.1, d—denotes the defuzzification method.

**Table 10:** The global scores of negotiation packages evaluated through OF-H1 for various sets of alternatives under consideration ($N_{1}$ and $N_{2}$).

Packages	OF-H1 $\;\mathrm{for}\;N_{1}\;(15)$		OF-H1 $\;\mathrm{for}\;N_{2}\;(7176)$	
	OF-H1	Rank	OF-H2	Rank
P1	0.044	15	0.147	15
P2	0.373	7	0.440	7
P3	0.191	12	0.278	12
P4	0.176	13	0.264	13
P5	0.306	10	0.381	10
P6	0.408	5	0.471	5
P7	0.459	4	0.517	4
P8	0.374	6	0.442	6
P9	0.116	14	0.211	14
P10	0.225	11	0.308	11
P11	0.345	9	0.415	9
P12	0.365	8	0.433	8
P13	0.560	2	0.607	2
P14	0.473	3	0.529	3
P15	0.679	1	0.713	1

Source: Own.

## Data Availability

Not applicable (for secondary data analysis, see [17]).

## Abbreviations

The following abbreviations are used in this manuscript:

MCDM	multiple criteria decision-making methods
FMCDM	fuzzy multiple criteria decision-making methods
DR	Direct Rating
AHP	Analytic Hierarchy Process
TOPSIS	Technique for Order Preferences by Similarity to Ideal Solution
DM	Decision maker
MARS	Measuring Attractiveness near Reference Situations
UTA	UTilités Additives
ELECTRE	ÉLimination Et Choix Traduisant la Realité
VIKOR	Serb. Vlse Kriterijumska Optimizacija i Kompromisno Resenje
OFN	Oriented Fuzzy Number
TrOFN	Trapezoidal Oriented Fuzzy Number
*H1*	Hellwig 1 method
*H2*	Hellwig 2 method
*OF-H1*	Hellwig 1 method based on oriented fuzzy numbers
*OF-H2*	Hellwig 2 method based on oriented fuzzy numbers
OF-SAW	Oriented Fuzzy SAW
OF-TOPSIS	Oriented Fuzzy TOPSIS
BATNA	Best Alternative to Negotiation Agreement
FOTE	full, open, truthful exchange
*PIS*	positive ideal solution
*NIS*	negative ideal solution
NOS	Numerical Order Scale
